# Analysis of the mechanisms regulating the expression of isoprenoid biosynthesis genes in hydroponically-grown *Nicotiana benthamiana* plants using virus-induced gene silencing

**DOI:** 10.1038/s41598-018-32901-5

**Published:** 2018-10-04

**Authors:** Go Atsumi, Uiko Kagaya, Noriko Tabayashi, Takeshi Matsumura

**Affiliations:** 1 National Institute of Advanced Industrial Science and Technology, 2-17-2-1, Tsukisamuhigashi, Toyohira-ku, Sapporo, Hokkaido 062-8517 Japan; 2 Plant Biotechnology Center, Hokusan Co. Ltd, 27-4, Kitanosato, Kitahiroshima, Hokkaido 061-1111 Japan

## Abstract

Secondary metabolites in plants play important roles in defence against biotic and abiotic stresses. Although the biosynthesis pathways of secondary metabolites have been extensively studied, the regulatory mechanism of gene expression involved in these pathways remains poorly understood. In this study, we develop a virus-induced gene silencing (VIGS) system that enables a rapid analysis of the regulatory mechanism of genes involved in the biosynthesis of isoprenoids, one of the largest groups in secondary metabolites, using hydroponically-grown *Nicotiana benthamiana*. Using VIGS, we successfully reduced the transcript levels of *3-hydroxy-3-methylglutaryl-CoA reductase 1* (*HMGR1*), *cycloartenol synthase 1* (*CAS1*), *sterol side chain reductase 2* (*SSR2*) and *S-adenosyl-L-Met-dependent C-24 sterol methyltransferase 1* (*SMT1*) in leaf, stem and root tissues in approximately 2 weeks. We identified novel feedback and feed-forward regulation of isoprenoid biosynthesis genes when *CAS1*, which encodes a key enzyme involved in the biosynthesis of sterols and steroidal glycoalkaloids, was down-regulated. Furthermore, the regulation of these genes differed among different tissues. These results demonstrate that our system can rapidly analyse the regulatory mechanisms involved in the biosynthesis of secondary metabolites.

## Introduction

Secondary metabolites in plants play important roles in defence against biotic stresses, such as herbivores and pathogens, and against abiotic stress, such as UV light^1^. Secondary metabolites also function to attract pollinators and animal vectors involved in seed dispersal, and are also used by humans as various chemicals, such as dyes, flavours, fragrances, medicines and insecticides^1^. Until now, more than 100,000 secondary metabolites have been identified in plants, and the variety of these metabolites in plants depends on the plant species^1^. Biosynthesis pathways of secondary metabolites have been extensively studied, and many biosynthesis enzymes have been identified. The biosynthesis and accumulation of secondary metabolites are regulated in an organ-, tissue- and cell-specific manner^2–4^. Transcriptional, translational and post-translational regulations are important to regulate each metabolic reaction^5^.

Feedback and feed-forward regulation of gene transcription is important to fine-tune the level of each metabolite^6–12^. The overexpression of genes or their knockdown using RNA interference (RNAi) has been used to investigate the regulation of gene expression. However, the generation of transgenic plants with altered gene expression levels is very laborious and time-consuming. Another obstacle in the generation of these transgenic plants is the deleterious effects on plant growth due to the manipulation of metabolic genes^13,14^. Although utilising cell culture systems, such as tobacco BY-2, can overcome some of these problems^9^, undifferentiated cells do not represent the physiology of differentiated cells. By contrast, virus-induced gene silencing (VIGS) technology can be used to manipulate transient gene expression in intact tissues. In VIGS, target transcripts are transiently degraded in a homology-dependent manner using a virus vector carrying a partial fragment of the target gene. VIGS does not require the generation of transgenic plants and has been used to suppress the expression of target genes in many plant species, including herbs and wood plants^15^. VIGS has been successfully used to study the function of genes involved in the biosynthesis of flavonoids in soybean (*Glycine max*)^16,17^ and steroidal glycoalkaloids in tomato (*Solanum lycopersicum*)^18–20^.

Isoprenoids constitute one of the largest groups of secondary metabolites; more than 50,000 isoprenoids have been identified until now^21^. In this study, we developed tobacco rattle virus (TRV)-based VIGS system to analyse the regulatory mechanisms of genes involved in the isoprenoid biosynthesis pathways in tobacco (*Nicotiana benthamiana*). We have selected a hydroponic culture system of *N. benthamiana*, which is suitable for the analysis of intact underground tissues and facilitates the control of factors affecting metabolite accumulation, such as the nutritional status. We demonstrated the successful down-regulation of genes including *3-hydroxy-3-methylglutaryl-CoA reductase 1* (*HMGR1*), *cycloartenol synthase 1* (*CAS1*), *sterol side chain reductase 2* (*SSR2*) and *S-adenosyl-L-Met-dependent C-24 sterol methyltransferase 1* (*SMT1*) in leaf, stem and root tissues. Using this experimental system, we identified novel feedback and feed-forward regulation of isoprenoid biosynthesis genes, and the differential regulation of these genes in different tissues.

## Results

### Comparison of VIGS efficiency between soil and hydroponic culture

**Figure 1:**
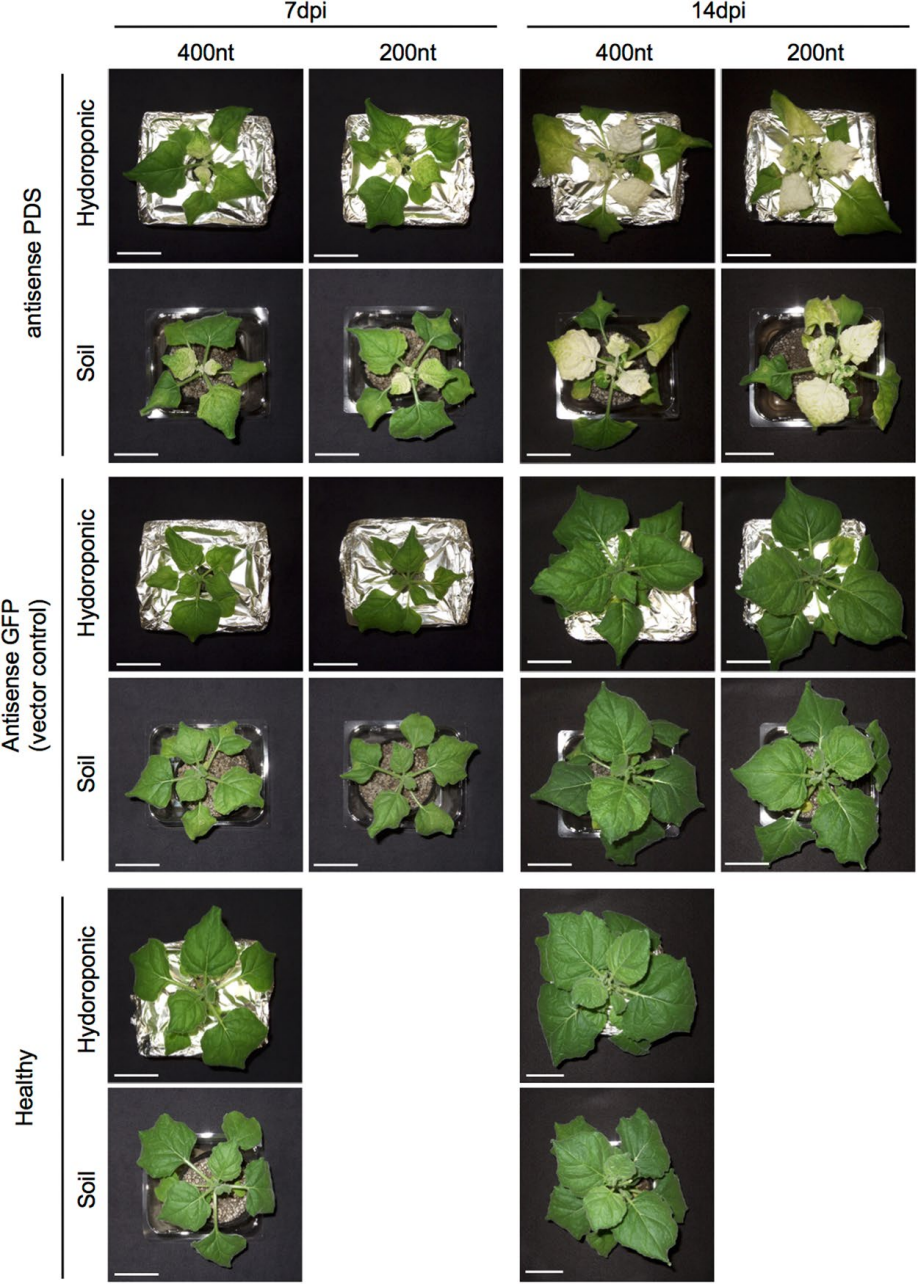
Knockdown of phytoene desaturase (*PDS*) gene using VIGS in hydroponically- or soil-grown *Nicotiana benthamiana* plants. Antisense partial fragment (400 or 200 nt) of *PDS* coding region was expressed in TRV vector. As a vector control, antisense partial fragment (400 or 200 nucleotides) of *GFP* gene was expressed. Photographs were taken at 7 and 14 days post-inoculation (dpi). Scale bar = 5 cm.

Since growth conditions, such as nutrient levels, affect the efficiency of RNA silencing^22,23^, we compared the efficiency of VIGS between hydroponically- and soil-grown plants of *N. benthamiana* using the *phytoene desaturase* (*PDS*) gene; this gene is widely used for the evaluation of the VIGS efficiency, as its knockdown results in the bleaching of plant tissues, such as leaves, which is easy to recognize visually^24,25^. For comparison, we cloned a 400-nt or 200-nt antisense sequence of the *PDS* coding region into the TRV vector (TRV/*asNbPDS400*, *asNbPDS200*). No striking differences were observed between hydroponically- and soil-grown plants, regardless of whether they were healthy or virus-infected (Fig. 1). As reported in previous studies^26,27^, plants inoculated with TRV/*asNbPDS400* and TRV/*asNbPDS200* began to show bleaching of leaves within 7 days post-infection (dpi), and extensive bleaching was observed within 14 dpi in soil-grown plants (Fig. 1). No marked differences were detected in the efficiency of VIGS of *PDS* between soil- and hydroponically-grown plants. These results suggest that VIGS is effectively induced in our hydroponic system.

### Cloning of *N. benthamiana* isoprenoid biosynthesis genes

**Figure 2:**
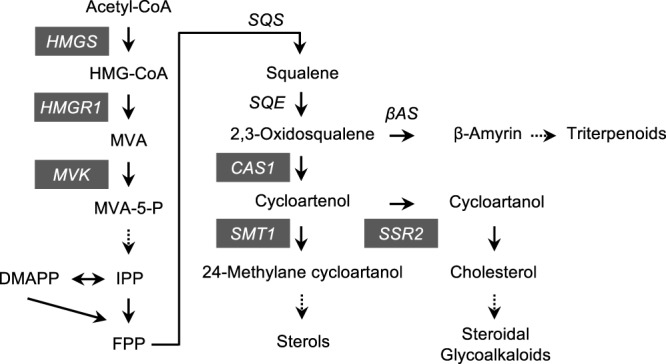
Schematic representation of isoprenoid biosynthesis pathway. HMG-CoA, 3-hydroxy-3-methyl-glutaryl-CoA; MVA, mevalonic acid; MVA-5-P, mevalonic acid-5-phosphate; IPP, isopentenyl diphosphate; DMAPP, dimethylallyl diphosphate; FPP, farnesyl diphosphate; HMGS, 3-hydroxy-3-methylglutaryl-CoA synthase; HMGR1, hydroxy-3-methylglutaryl-CoA reductase 1; MVK, mevalonate kinase; SQS, squalene synthase; SQE, squalene epoxidase; βAS, β-amyrin synthase; CAS1, cycloartenol synthase 1; SSR2, sterol side chain reductase 2; SMT1, S-adenosyl-L-Met-dependent C-24 sterol methyltransferase 1. Solid and dashed arrows indicate single-step and multi-step reactions, respectively. Genes highlighted in grey represent those used in this study.

We cloned six isoprenoid biosynthesis genes including, *3-hydroxy-3-methylglutaryl-CoA synthase* (*HMGS*), *HMGR1*, *mevalonate kinase* (*MVK*), *CAS1*, *SSR2* and *SMT1* using genome sequence draft of *N. benthamiana* (Sol Genomics Network database, https://solgenomics.net/) (Fig. 2). We identified two closely-related sequences of each gene, which is not inconsistent with allotetraploidy of *N. benthamiana*^28^. To differentiate between the two copies of a gene, we used ‘a’ and ‘b’ as suffixes (see Supplementary Table S1). The lengths of open reading frames (ORFs) of each gene, and nucleotide identities between the two copies of a gene are summarised in Supplementary Tables S1 and S2, respectively.

BLAST search identified two *HMGS* candidates, Niben101Scf01111g01003 (*NbHMGSa*) and Niben101Scf01729g01015 (*NbHMGSb*), which were similar to the tomato ortholog, *SlHMGS*, as indicated by phylogenetic analysis (see Supplementary Fig. S1)^29^. BLAST search using the *N. tabacum* orthologs of *HMGR*, *NtHMGR1* and *NtHMGR2*^30^, revealed several candidate clones including truncated sequences. We determined three candidates, Niben101Scf09686g00013 (*NbHMGR1a*), Niben101Scf13180g01003 (*NbHMGR1b*) and Niben101Scf02203g05002 (*NbHMGR2a*). Phylogenetic analysis revealed that *NbHMGR1a* and *NbHMGR1b* were more closely related to *NtHMGR1*^30^ and *StHMGR1*^31^, respectively, than to *NtHMGR2*^30^ and *StHMGR2*^31^, which were close to *NbHMGR2a* (see Supplementary Fig. S1). BLAST search using *Arabidopsis thaliana AtMVK*^32^ and keyword search identified two genes, Niben101Scf25893g00005 (*NbMVKa*) and Niben101Scf00370g03023 (*NbMVKb*) (see Supplementary Fig. S1). A partial sequence of *NbCAS1* (Niben101Scf16532g01001: former ID was NbS00021029g0013), which we refer to as *NbCAS1a*, has been isolated previously^33^. In addition to *NbCAS1a*, we identified a novel *CAS1* allele, Niben101Scf08080g00009 (*NbCAS1b*), and determined the sequences of both ORFs. Phylogenetic analysis suggested that *NbCAS1a* and *NbCAS1b* were orthologous to *NtCAS1*^33^ (see Supplementary Fig. S1). BLAST search using *SlSSR2*^34^ identified four closely-related sequences, Niben101Scf03969g04003 (*NbSSR2a*), Niben101Scf00271g04029 (*NbSSR2b*), Niben101Scf02156g03023 and Niben101Scf04964g02005; sequences of these genes had nucleotide identities, 87.4%, 86.9%, 79.9% and 77.2% with *SlSSR2*, respectively. Phylogenetic analysis suggested that *NbSSR2a* and *NbSSR2b* were more closely related to *StSSR2* and *SlSSR2*, respectively, than to *StSSR1* and *SlSSR1* (see Supplementary Fig. S1). BLAST search using *NtSMT1*^35,36^ revealed two candidate genes, Niben101Scf13874g01006 (*NbSMT1a*) and Niben101Scf03085g05002 (*NbSMT1b*), which were closely related to *NtSMT1-1* (U81312) and *NtSMT1-2* (AF053766), respectively (see Supplementary Fig. S1).

### Knockdown of isoprenoid biosynthesis genes using VIGS

We constructed TRV vectors to down-regulate *NbHMGR1*, *NbCAS1*, *NbSSR2* and *NbSMT1*. We cloned 400 nt antisense fragments of these genes in the TRV vectors to target both transcript copies (a and b) of each gene (see Methods). The knockdown of *NbHMGR1*, *NbSSR2* and *NbSMT1* did not markedly affect plant growth in comparison to the vector control expressing antisense partial GFP fragment (*asGFP*) plants. By contrast, knockdown of *NbCAS1* expression gradually induced cell death along veins in leaf tissues within 7 dpi (see Supplementary Fig. S2).

Because secondary metabolites are often synthesised in a tissue-specific manner^3,37^, we analysed the knockdown efficiency of various isoprenoid biosynthesis genes in leaves, stems and roots via real-time PCR. Both transcripts of each gene (a and b) were detected simultaneously in these tissues, except for *NbSMT1*. As shown in Fig. 1, VIGS against *PDS* gene was extensively and uniformly induced in leaves at 14 dpi. Therefore, we investigated the level of each transcript by real-time PCR at 15 dpi.

**Figure 3:**
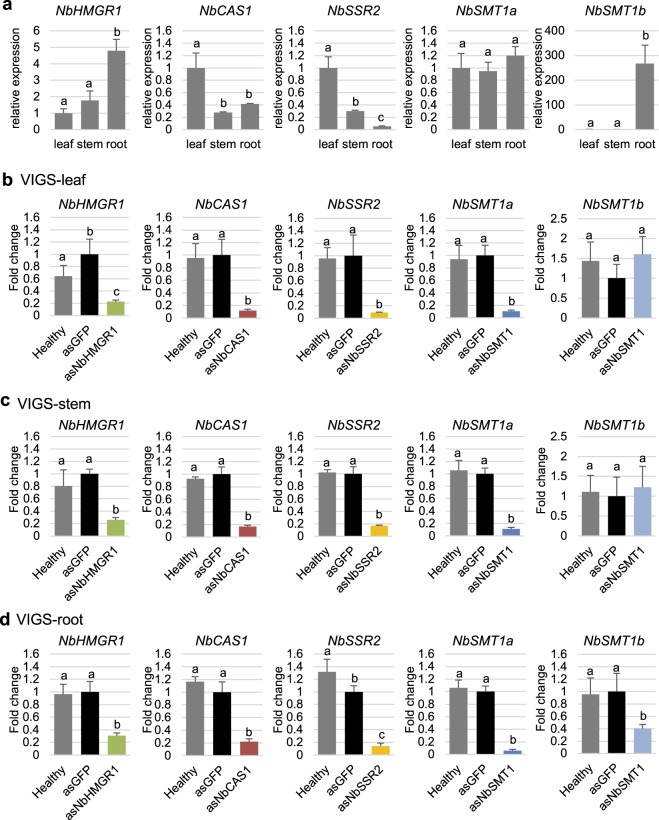
Expression level of each isoprenoid biosynthesis gene targeted by VIGS in hydroponically-grown *N. benthamiana*. Each partial antisense sequence of *NbHMGR1* (*asNbHMGR1*), *NbCAS1* (*asNbCAS1*), *NbSSR2* (*asNbSSR2*), *NbSMT1* (*asNbSMT1*) or *GFP* (*asGFP*, vector control) was expressed in TRV vector. Total RNA was extracted from leaf (**a**,**b**), stem (**a**,**c**) or root (**a**,**d**) tissues at 15 dpi and used to synthesise cDNAs for real-time PCR analysis. The expression level of each gene was normalised relative to that of *NbEF1α*. Fold changes in expression level are indicated relative to the vector control (*asGFP*). Error bars indicate standard deviation of four biological replicates. Statistical analyses were conducted using the Tukey–Kramer test^50^. Different letters above bars indicate statistically significant differences (*P* < 0.05).

We compared the basal level of each gene among leaf, stem and root tissues in healthy plants used for negative control. Real-time PCR analysis showed that *NbHMGR1* expression in leaves and stems was significantly lower than that in roots, respectively (Fig. 3a). The expression of *NbCAS1* and *NbSSR2* in leaves was higher than that in stems and roots (Fig. 3a). Whereas no significant difference was observed in *NbSMT1a* expression, *NbSMT1b* expression in leaves and stems was >250-fold lower than that in roots (Fig. 3a).

Compared with the control, transcript levels of *NbHMGR1*, *NbCAS1*, *NbSSR2* and *NbSMT1a* was reduced to approximately 23%, 12%, 9% and 11%, respectively, in leaf tissues (Fig. 3b), 26%, 17%, 17% and 11%, respectively, in stem tissues (Fig. 3c) and 31%, 22%, 14% and 6%, respectively, in root tissues by VIGS (Fig. 3d). The transcript level of *NbSMT1b* was reduced to approximately 40% in root tissues, although no significant decrease was observed in leaf and stem tissues (Fig. 3b–d), which might be due to low expression levels of *NbSMT1b* in leaf and stem tissues, as stated above (Fig. 3a). These results demonstrate that the TRV vectors effectively down-regulate isoprenoid biosynthesis genes in leaf, stem and root tissues of *N. benthamiana*.

To investigate whether the metabolite composition was changed in *NbHMGR1-*, *NbCAS1-*, *NbSSR2*- and *NbSMT1*-silenced plants, leaf extracts were prepared from each plant inoculated with TRV/*asNbHMGR1*, TRV/*asNbCAS1*, TRV/*asNbSSR2* or TRV/*asNbSMT1* at 16 days after inoculation, and metabolites in the extract were analysed by gas chromatography/mass spectrometry (GC/MS). There was no striking difference in metabolite levels such as phytosterols (campesterol and stigmasterol) in *NbHMGR1*-silenced plants compared with plants inoculated with the vector control (TRV/*asGFP*) (see Supplementary Fig. S3). *NbCAS1*-knock-down increased the level of 2,3-oxidosqualene and reduced the levels of cholesterol, campesterol and stigmasterol (see Supplementary Fig. S3). *NbSSR2*-knock-down reduced the level of cholesterol as reported in *SSR2*-silenced potato and tomato^34^ (see Supplementary Fig. S3). *NbSMT1*-knock-down increased the level of cholesterol as reported in *A. thaliana smt1* mutant^14^, and reduced the levels of campesterol and stigmasterol (see Supplementary Fig. S3). These results indicated that our experimental system could successfully change metabolite composition in the isoprenoid pathway.

### Feedback and feed-forward regulation of isoprenoid biosynthesis pathways in *N. benthamiana*

**Figure 4:**
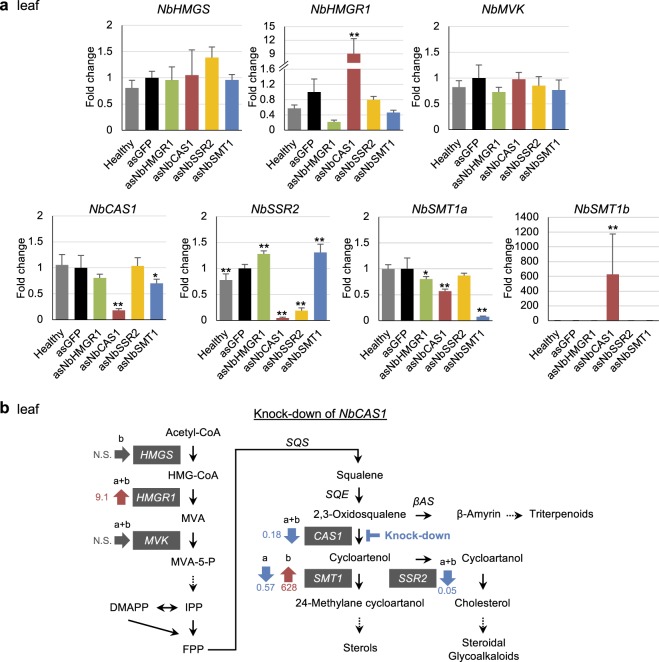
Feedback and feed-forward regulation in isoprenoid biosynthesis genes in *NbCAS1* knockdown leaves. (**a**) Real-time PCR analysis of isoprenoid biosynthesis genes in leaf tissues at 16 dpi with each TRV vector. The expression level of various genes was normalised relative to that of *NbEF1α*. Fold change is presented relative to the vector control (*asGFP*). Error bars indicate standard deviation of four biological replicates. Statistical analyses were conducted using the Dunnett method. Data from vector control were used as a control for statistical analysis. *P < 0.05; **P < 0.01. (**b**) Schematic representation of fold changes in transcript levels of genes relative to the vector control (*asGFP*) in the *NbCAS1* knockdown leaves in isoprenoid biosynthesis pathway. Abbreviations of genes are described in the legend of Fig. 2. N.S. = not significant.

**Figure 5:**
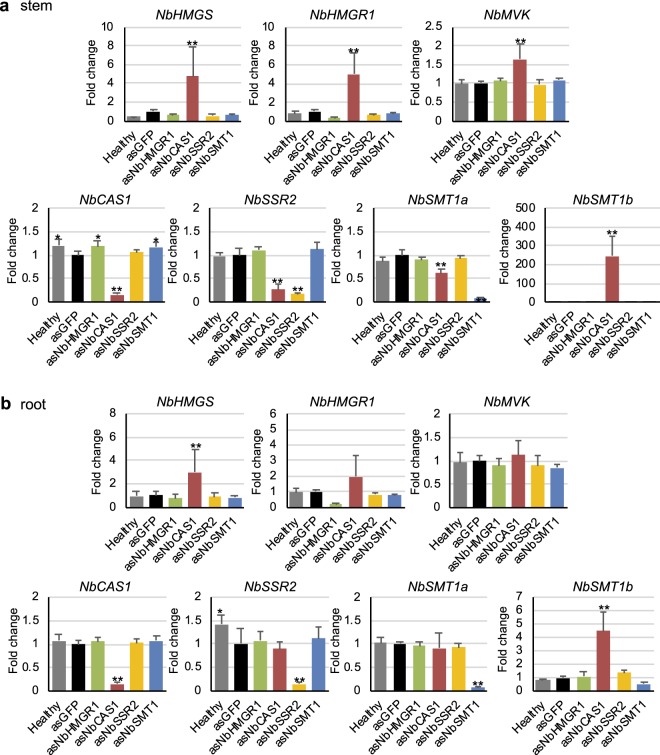
Feedback and feed-forward regulation in isoprenoid biosynthesis genes in *NbCAS1* knockdown stems and roots. Real-time PCR analysis of isoprenoid biosynthesis genes in the stem (**a**) and root (**b**) tissues at 16 dpi with each TRV vector. The expression level of various genes was normalised relative to that of the *NbEF1α* gene. Fold change is represented relative to the vector control (*asGFP*). Error bars indicate standard deviation of four biological replicates. Statistical analyses were conducted using the Dunnett method. Data from vector control were used as a control for statistical analysis. *P < 0.05; **P < 0.01.

We analysed feedback and feed-forward regulation of isoprenoid biosynthesis genes using VIGS. The expression of *NbHMGR1*, *NbCAS1*, *NbSSR2* and *NbSMT1* was transiently suppressed by VIGS, and their effect on expression levels of *NbHMGS* (*NbHMGSb* detected), *NbHMGR*, *NbMVK* (both *NbMVKa* and *NbMVKb*), *NbCAS1*, *NbSSR2* and *NbSMT1* was analysed in leaf tissues. The suppression of *NbCAS1* significantly altered the expression of genes both upstream and downstream of *NbCAS1* in the isoprenoid biosynthesis pathway. For upstream genes, *NbCAS1* knockdown significantly increased *NbHMGR1* expression in leaf tissues; however, the expression of *NbHMGS* and *NbMVK* was not affected (Fig. 4a). For downstream genes, *NbCAS1* knockdown significantly decreased the expression of *NbSSR2* and *NbSMT1a* to approximately 5% and <60%, respectively (Fig. 4a). By contrast, the expression of *NbSMT1b* was significantly increased (>600-fold) in leaves of *NbCAS1* knockdown plants (Fig. 4a), suggesting that *NbSMT1a* and *NbSMT1b* are regulated antagonistically (Fig. 4b). In stem tissues of *NbCAS1* knockdown plants, the expression of *NbHMGR1*, *NbSMT1b*, *NbHMGS* and *NbMVK* was up-regulated, whereas that of *NbSSR2* and *NbSMT1a* was down-regulated (Fig. 5a, Supplementary Fig. S4). In root tissues of *NbCAS1* knockdown plants, the expression of *NbHMGS* and *NbSMT1b* was up-regulated; however, no significant differences were observed in the expression of other genes (Fig. 5b, Supplementary Fig. S4). In the case of *NbSSR2*, no difference in its transcript level in roots of *NbCAS1* knockdown plants may be explained by a lower basal level of expression of *NbSSR2* in roots compared with leaves and stems (Figs 3a and 5b). These results suggest that the down-regulation of *NbCAS1* expression enhances the upstream pathway and weakens the downstream pathway at the transcriptional level in leaf and stem tissues.

**Figure 6:**
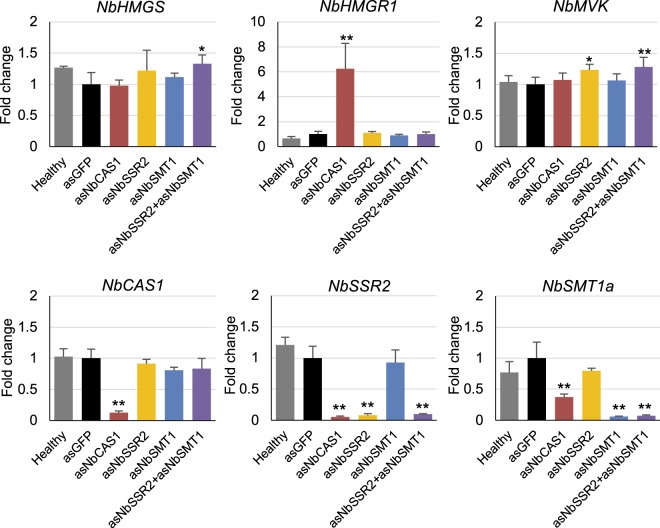
Effect of simultaneous knockdown of *NbSSR2* and *NbSMT1* on the expression of isoprenoid biosynthesis genes. Real-time PCR analysis of isoprenoid biosynthesis genes in leaves at 17 dpi with each TRV vector. The expression level of various genes was normalised to that of *NbEF1α*. Fold change is represented relative to the vector control (*asGFP*). Error bars indicate standard deviation of four biological replicates. Statistical analyses were conducted using the Dunnett method. Data from vector control (*asGFP*) were used as a control for statistical analysis. *P < 0.05; **P < 0.01.

CAS1 converts 2,3-oxidosqualene to cycloartenol^38^, which is then converted into cycloartanol or 24-methylane cycloartanol by SSR2^34^ or SMT1^14^, respectively. (Fig. 2). We showed that *NbCAS1* knockdown up-regulated *NbHMGR1* expression; however, knockdown of *NbSMT1* or *NbSSR2* did not increase *NbHMGR1* expression (Fig. 4a). We investigated whether simultaneous down-regulation of *NbSSR2* and *NbSMT1* up-regulated *NbHMGR1* expression. We constructed a TRV vector carrying 200-nt antisense sequences of *NbSSR2* and *NbSMT1* (TRV/*asNbSSR2* + *asNbSMT1*), as 200-nt antisense sequences were shown sufficient for the induction of VIGS against *PDS* gene (Fig. 1). In leaves infected with TRV/*asNbSSR2* + *asNbSMT1*, the expression of *NbSSR2* and *NbSMT1a* was reduced to approximately 9.8% (*NbSSR2ab*) and 7.3% (*NbSMT1a*); these levels were comparable with those in single *asNbSSR2* knockdown plants (decreased to 8.1%) or *asNbSMT1* (decreased to 5.9%), respectively (Fig. 6). Expression analyses indicated that leaves infected with TRV/*asNbSSR2* + *asNbSMT1* did not show up-regulation of *NbHMGR1* expression (Fig. 6).

## Discussion

We could successfully down-regulated the expression of isoprenoid biosynthesis genes (Fig. 3), and present evidence for some transcriptional feedback and feed-forward regulation using VIGS in hydroponically-grown *N. benthamiana* (Figs 4 and 5). We showed that *NbCAS1* knockdown up-regulated *NbHMGR1* expression; however, simultaneous down-regulation of *NbSSR2* and *NbSMT1* did not increase the level of *NbHMGR1* transcripts (Figs 4 and 6), implying that a decrease in the level of cycloartenol triggers the increase in *NbHMGR1* expression. This possibility is also mentioned in a previous study using transgenic *N. tabacum* lines overexpressing *SMT1*; the overexpression of *SMT1* in *N. tabacum* reduces the level of cycloartenol compared with wild-type plants and increases *HMGR1* expression^6^. However, another recent study has shown that the basal level of cycloartenol accumulation is often below the limit of detection in *N. benthamiana*^33^. Therefore, a decrease in cycloartenol itself might not trigger the feedback regulation. GC/MS analysis indicated that 2,3-oxidosqualene was extensively accumulated in *NbCAS1*-knock-down plants but not in control plants (see Supplementary Fig. S3). Therefore, the feed-back regulation on *HMGR1* might be triggered to metabolize the over-accumulated 2,3-oxidosqualene. Another possibility is that a transient decrease in *NbSSR2* and *NbSMT1* expression may insufficiently decrease the level of downstream metabolites required for feedback regulation, although *NbCAS1* knockdown rapidly decreases the level of cycloartenol and downstream metabolites that use cycloartenol as a substrate.

Up-regulated expression of *NbHMGR1* in leaves and stems of *NbCAS1* knockdown plants of *N. benthamiana* plants is inconsistent with a previous study using tobacco BY2 cells showing unchanged *NtHMGR1* transcript level and reduction in *NtHMGR2* transcripts in *CAS1* knockdown BY2 cells^9^. These contradictory results might originate from differences between tissue types (leaf and stem) and cell culture or the degree of knockdown; *CAS1* expression decreased to 17% in leaves and 22% in stems in this study (Figs 4a and 5a) but to 65% in BY2 cell cultures^9^.

Feedback regulation of *HMGR* is also observed in pharmacological inhibition of enzymes or knockdown of genes regulating squalene metabolism. Treatment with either squalestatin [a squalene synthase (SQS) inhibitor] or terbinafine [a squalene epoxidase (SQE) inhibitor] increases the enzymatic activity of HMGR in tobacco BY2 cell cultures^7^ and *A. thaliana*^12,39^. By contrast, there are controversies over the transcriptional regulation. Wentzinger *et al*. have shown that treatment with squalestatin, but not terbinafine, increases the mRNA level of *NtHMGR*^7^. Kobayashi *et al*. have shown that the expression of *AtHMGR1* is up-regulated by squalestatin and down-regulated by terbinafine treatment in *A. thaliana* carrying *35S::ADS*^12^. By contrast, Nieto *et al*. have shown that neither squalestatin nor terbinafine treatment changes the mRNA level of *AtHMGR1* or *AtHMGR2* in *A. thaliana*^39^. These contradictory results may be due to differences in experimental conditions^12^ and/or due to lack of coordination between gene expression level and enzyme activity^39^. Singh and colleagues have shown that knockdown of *SQS* via VIGS up-regulates the expression of upstream genes, including *HMGR*, and suppresses the expression of downstream genes in *Withania somnifera*^10^. Under biotic stress condition, Chappell *et al*. have shown that pathogen elicitor upregulates HMGR1 activity and downregulates SQS activity^40^. Collectively, these reports indicate that SQS is a major control point in isoprenoid pathway. As knockdown of *CAS1* triggered feed-back regulatory response on *HMGR1* expression, CAS1 might also be an important control point, although it is unknown that the feed-back regulation also occurred on HMGR1 enzymatic activity.

We showed that *NbSSR2* expression was significantly decreased in leaves and stems of *NbCAS1* knockdown plants (Figs 4a and 5a). The GAME9 protein, an APETALA2/Ethylene Response Factor, regulates the expression of both *SSR2* and *CAS1* in tomato and potato leaves^19^. Co-expression analysis indicates that *SlSSR2* is co-expressed with 13 sterol metabolism-related genes, including *SlCAS1* in tomato^20^. It is possible that *NbCAS1* and *NbSSR2* are also transcriptionally co-regulated in *N. benthamiana*. Elucidation of the mechanism that explains the reduction in *NbSSR2* expression in *NbCAS1* knockdown plants will provide new insights into the transcriptional regulation of *NbSSR2*.

Additionally, we showed that *NbSMT1a* and *NbSMT1b* were under antagonistic regulation; *NbSMT1a* was down-regulated, whereas *NbSMT1b* was significantly up-regulated in *NbCAS1* knockdown plants (Figs 4a and 5a). The transcript level of *NbSMT1a* was not significantly different among leaf, stem and root tissues, whereas that of *NbSMT1b* was significantly lower in leaves and stems than in roots (Fig. 4), indicating their differential transcriptional regulation. Because *N. benthamiana* is an allotetraploid^28^, *NbSMT1a* and *NbSMT1b* may be homoeologs^41^. Although models for regulatory relationships among homoeologous genes have been proposed^42^, the information available is insufficient. *N. benthamiana* will serve as an excellent model for elucidating regulatory differences between homoeologs.

In summary, we could successfully down-regulate the isoprenoid biosynthesis genes, and could reveal novel feed-back and feed-forward regulations using hydroponically-grown *N. benthamiana* in approximately two weeks. Our VIGS system will be a powerful tool to elucidate gene function and regulatory gene networks involved in the biosynthesis of secondary metabolites, especially if knockdown of the target gene has deleterious effects on plant growth.

## Methods

### Plant growth conditions

*N. benthamiana* was grown hydroponically in a nutrient solution (Otsuka hydroponic composition, Otsuka Chemical Co., Ltd., Osaka, Japan) or in soil at 24 °C and 16 h light/8 h dark. The nutrient solution (pH 6.0) used for hydroponic culture contained N (175.1 ppm), P_2_O_2_ (80 ppm), K_2_O (272.7 ppm), CaO (153.3 ppm), MgO (40 ppm), MnO (1.6 ppm), B_2_O_3_ (1.6 ppm), Fe (3.51 ppm), Cu (0.032 ppm), Zn (0.084 ppm) and Mo (0.0329 ppm).

### Isolation of genes encoding biosynthesis enzymes of isoprenoids

*N. benthamiana* tissues were homogenised in liquid nitrogen. Total RNA was isolated using the acid guanidinium thiocyanate–phenol–chloroform (AGPC) extraction method^43^, followed by purification with a FARB minicolumn (Favorgen Biotech Corp., Ping-Tung, Taiwan). Total RNA was digested with Turbo DNase (Thermo Fisher Scientific, Waltham, MA, USA) and reverse transcribed using random hexamer or oligo-dT by PrimeScript reverse transcriptase (TaKaRa Bio, Kusatsu, Japan), according to the manufacturer’s instructions.

Primers for the isolation of putative ORFs of *N. benthamiana* were designed using sequence information of *N. benthamiana* draft genome v1.0.1 available for predicted cDNAs at Sol Genomics Network (https://solgenomics.net/) by conducting BLAST and keyword searches (see Supplementary Table S3). Each amplified fragment was cloned into the pGEM-T Easy vector (Promega, Fitchburg, WI, USA) or pCR4Blunt-TOPO vector (Thermo Fisher Scientific). Each consensus sequence was determined from at least three plasmid clones. We also conducted 5′ rapid amplification of cDNA ends (RACE) using a GeneRacer Kit (Thermo Fisher Scientific), according to the manufacturer’s instructions, and determined the putative start codon of each fragment.

### Phylogenetic analysis

Sequence alignments were conducted by using MUSCLE^44^, and a maximum likelihood tree was inferred using the MEGA7 package^45^. Models for nucleotide substitution and rates among sites were determined using MEGA7. For all genes, the Tamura 3-parameter model^46^ was used as the nucleotide substitution model, and a discrete gamma distribution was used to model evolutionary rate differences among sites. Only for *CAS1*, the rate variation model allowed some sites to be evolutionarily invariable. The significance of the nodes was estimated with 1,000 bootstrap replicates.

### Preparation of plasmid constructs

pTRV1 (stock# CD3-1039) and pTRV2-MCS (stock# CD3-1040) were obtained from the Arabidopsis Biological Resource Center (ABRC), Ohio, USA. VIGS constructs were designed using SGN VIGS Tool (vigs.solgenomics.net) with modifications. Each fragment was PCR amplified using primers described in Supplementary Table S4, followed by digestion with *Eco*RI and *Bam*HI. The digested fragments were cloned into pTRV2-MCS digested with *Eco*RI and *Bam*HI.

### Virus inoculation

*Agrobacterium*-mediated inoculation was conducted as described previously^26,47–49^. *Agrobacterium tumefaciens* LBA4404 cells transformed with each construct were suspended in MES buffer [10 mM 2-(N-morpholino)ethane-sulfonic acid, 10 mM MgCl_2_ (pH 5.7)], and cell suspensions were adjusted to an optical density at 600 nm (OD_600_) of 0.5. Acetosyringone was added to the suspensions at a final concentration of 200 μM, followed by incubation at room temperature for 2–4 h. Suspensions of *Agrobacterium* carrying pTRV1 and pTRV2 carrying the target constructs were mixed in a 1:1 (v/v) ratio and infiltrated into *N. benthamiana* leaves using a needleless syringe.

### Real-time RT-PCR

The leaf, stem and root tissues of *N. benthamiana* were homogenised in liquid nitrogen. Total RNA was isolated from these tissue samples using the AGPC extraction method and then purified with a FARB minicolumn (Favorgen Biotech Corp.). Total RNA was digested with Turbo DNase (Thermo Fisher Scientific) and reverse transcribed using random hexamer by PrimeScript II reverse transcriptase (TaKaRa), according to the manufacturer’s instructions. Real-time PCR was performed using LightCycler 96 system (Roche Diagnostics, Basel, Switzerland). The reaction mixture (10 μl) contained FastStart Essential DNA Probes Master (Roche Diagnostics), 0.5 μM each of forward and reverse primers, 0.2 μM Universal ProbeLibrary Probe (Roche Diagnostics) and cDNA obtained by reverse transcribing 5–10 ng of total RNA. Samples were incubated for 10 min at 95 °C, followed by 45 cycles of 95 °C for 10 s and 60 °C for 30 s. Transcript levels of each gene were normalised to those of *NbEF1α* (GenBank accession number AY206004). Primers and probes were designed using Universal ProbeLibrary Assay Design Center (https://qpcr.probefinder.com/organism.jsp) and are listed in Supplementary Table S5.

### GC/MS analysis

Leaf extracts were prepared according to Itkin *et al*.^49^. Freeze-dried *N. benthamiana* leaves were disrupted into a fine powder by the use of the Multi-beads shocker MB701 (Yasui Kikai, Osaka, Japan). The extraction was conducted at 75 °C for 1 hour with 20 ml of chloroform-methanol (2:1[v/v]), followed by keeping the samples at room temperature for 1 hour. Extracts were dried by evaporation and were saponified at 90 °C for 1 hour in 20 ml 6% (w/v) KOH in methanol. After cooling of samples to RT, 12 ml *n*-hexane and 12 ml water were added, and the mixture was shaken for 30 s. After centrifugation, the *n*-hexane phase was collected. The aqueous phase was re-extracted twice by *n*-hexane. The collected *n*-hexane phases were evaporated and the residues re-suspended in 0.5 ml *n*-hexane. 5β-Cholestan-3α-ol (Sigma-Aldrich, MO, USA) was used as an internal standard, and *N*-Methyl-*N*-trimethylsililtrifluoroacatamide (Tokyo Chemical Industry, Tokyo, Japan) was used for trimethylsilylation. Samples mixed with 5β-Cholestan-3α-ol and *N*-Methyl-*N*-trimethylsililtrifluoroacatamide were incubated at RT for 30 min, and used for GC/MS analysis by a GCMS-QP2020 system with GC-2010plus (Shimazu, Kyoto, Japan). GC analysis was performed with Rxi-5SilMS (30 m × 0.25 mm, 0.25 μm film thickness) column (Shimazu). Helium (99.9999%) was used as carrier gas at a flow of 1.2 ml/min with constant linear velocity mode, 40 cm/s. The injection volume was 2.0 μl with splitless mode, and injector temperature was set at 250 °C. The column temperature was programmed as follows, 60 °C for 3 min, an increase of 30 °C/min to 220 °C, followed by an increase of 2 °C/min to 300 °C, ending with a hold at 300 °C for 10 min. The ion source temperature was kept at 230 °C, and the interface temperature at 320 °C. Mass spectra were taken at 70 eV, with a scan interval of 0.3 s and ions were monitored from 40 to 550 *m*/*z*.

### Nucleotide sequence accession number

The GenBank/ENA/DDBJ accession numbers for putative full-length ORF sequence of *HMGS*, *HMGR1*, *MVK*, *CAS1*, *SSR2* and *SMT1* are LC382272 (*NbHMGSa*), LC382273 (*NbHMGSb*), LC382274 (*NbHMGR1a*), LC382275 (*NbHMGR1b*), LC382276 (*NbHMGR2a*), LC382277 (*NbMVKa*), LC382278 (*NbMVKb*), LC382279 (*NbCAS1a*), LC382280 (*NbCAS1b*), LC382281 (*NbSSR2a*), LC382282 (*NbSSR2b*), LC382283 (*NbSMT1a*) and LC382284 (*NbSMT1b*).

## Electronic supplementary material

Supplementary Information

## Data Availability

The GenBank/ENA/DDBJ accession numbers for genes isolated in this study were described in Methods section. The data supporting the findings of this study are available within the manuscript or upon request.
