# Supplementary material for: Analysis of the mechanisms regulating the expression of isoprenoid biosynthesis genes in hydroponically-grown Nicotiana benthamiana plants using virus-induced gene silencing
Source: Sci Rep. 2018 Oct 4;8:14804. doi: 10.1038/s41598-018-32901-5 (PMC6172274; doi:10.1038/s41598-018-32901-5)
Supplement: Supplementary file 1 — Supplementary Information [file 41598_2018_32901_MOESM1_ESM.pdf]

Supplementary Information

Analysis of the mechanisms regulating the expression of isoprenoid biosynthesis genes in hydroponically-grown *Nicotiana benthamiana* plants using virus-induced gene silencing

Go Atsumi[1*], Uiko Kagaya[2], Noriko Tabayashi[2] and Takeshi Matsumura[1]

[1]National Institute of Advanced Industrial Science and Technology,
Tsukisamuhigashi 2-17-2-1, Toyohira-ku, Sapporo, Hokkaido 062-8517, Japan
[2]Plant Biotechnology Center, Hokusan Co., Ltd, Kitanosato 27-4,
Kitahiroshima, Hokkaido 061-1111, Japan.

[*]Correspondence and requests for materials should be addressed to Go Atsumi
(email: go-atsumi@aist.go.jp)

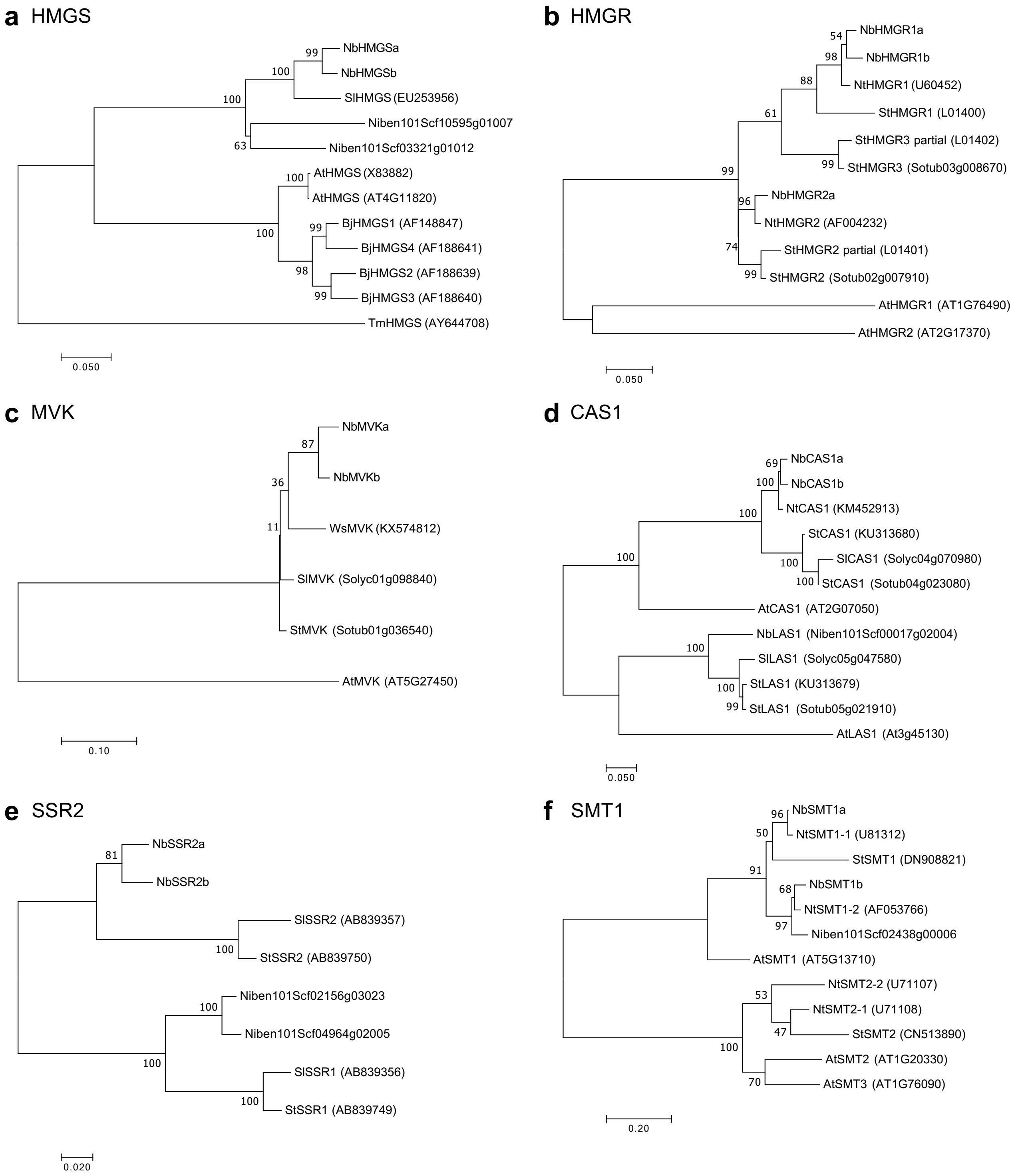

Supplementary Figure S1. Molecular phylogenetic analysis of isoprenoid biosynthetic genes isolated in this study. Putative full length ORF sequences determined in this study and from database were aligned by MUSCLE, and the maximum likelihood tree was inferred by MEGA7. The tree is drawn to scale, with branch lengths measured as the number of substitutions per site. The significance of the nodes was estimated with 1,000 bootstrap replicates. The GenBank accession number or gene ID for each gene are noted in parentheses after gene name. Genes started with Niben101Scf are obtained from draft genome sequence of *N. benthamiana* in Sol Genomics Network (https://solgenomics.net/) as described in main text. Nb = *Nicotiana benthamiana*, Nt = *Nicotiana tabacum*, Sl = *Solanum lycopersicum*, St = *Solanum tuberosum*, At = *Arabidopsis thaliana*, Bj = *Brassica juncea*, Tm = *Taxus x media*, Ws = *Withania sommifera*.

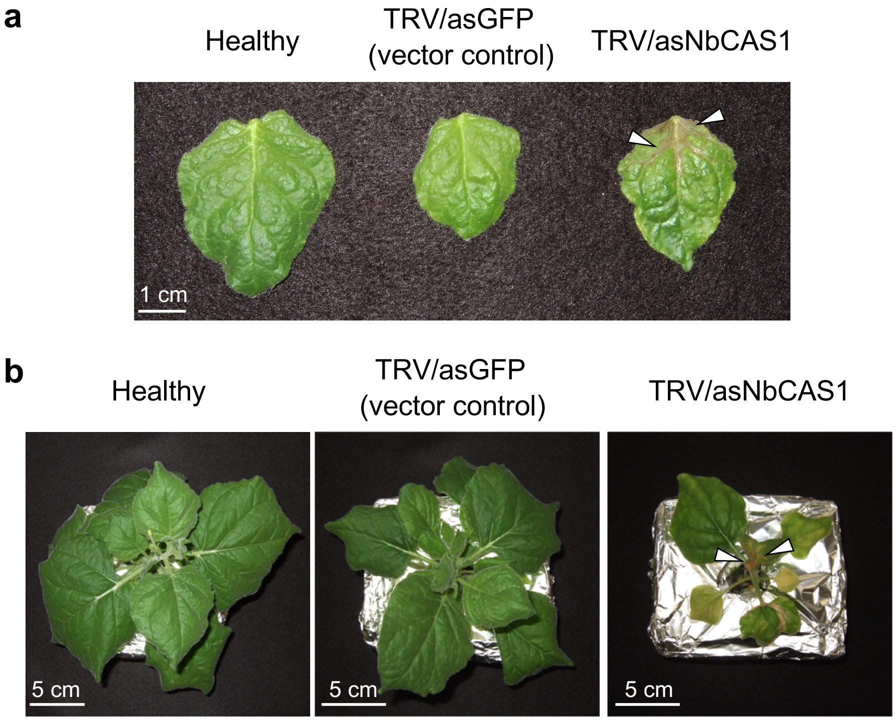

Supplementary Figure S2. Phenotype of *NbCAS1*-silenced plants by VIGS in *N. benthamiana*. Photographs of upper leaves (a) and of whole plants (b) were taken at 10 and 14 days after inoculation, respectively. White arrowheads indicate cell death-induced area.

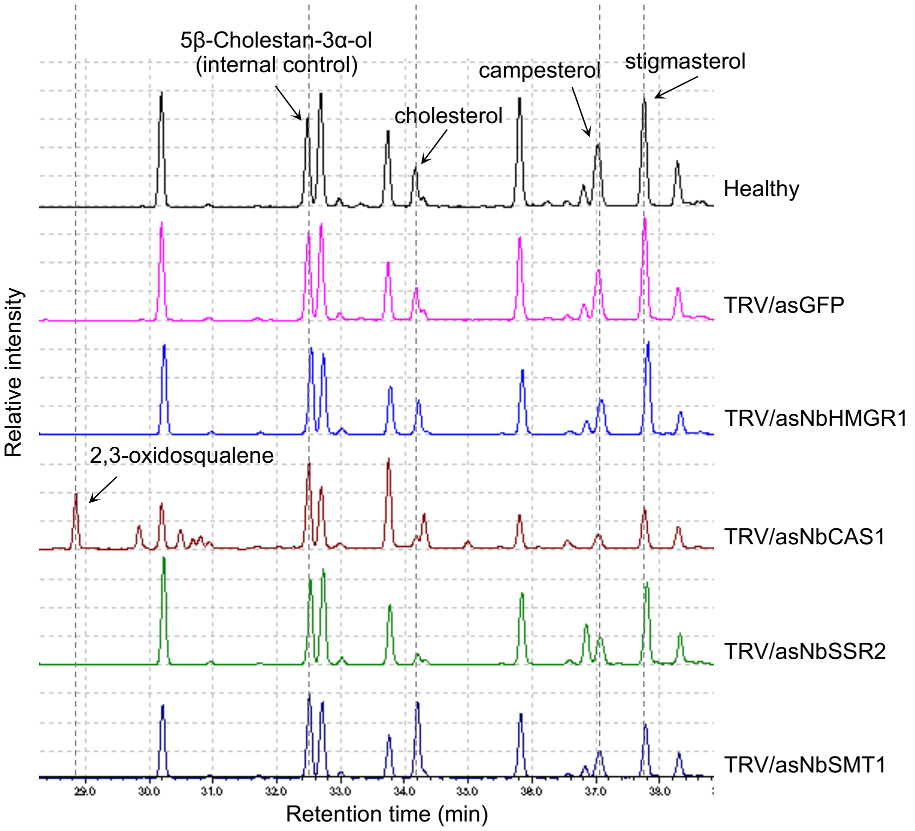

Supplementary Figure S3. Accumulations of metabolite in *NbHMGR1*, *NbCAS1*, *NbSSR2*, and *NbSMT1*-silenced *N.benthamiana*. Leaf extract was prepared from each plant inoculated with TRV/asNbHMGR1, TRV/asNbCAS1, TRV/asNbSSR2 or TRV/asNbSMT1 at 16 days after inoculation, and metabolites in the extract was analysed by GC/MS.

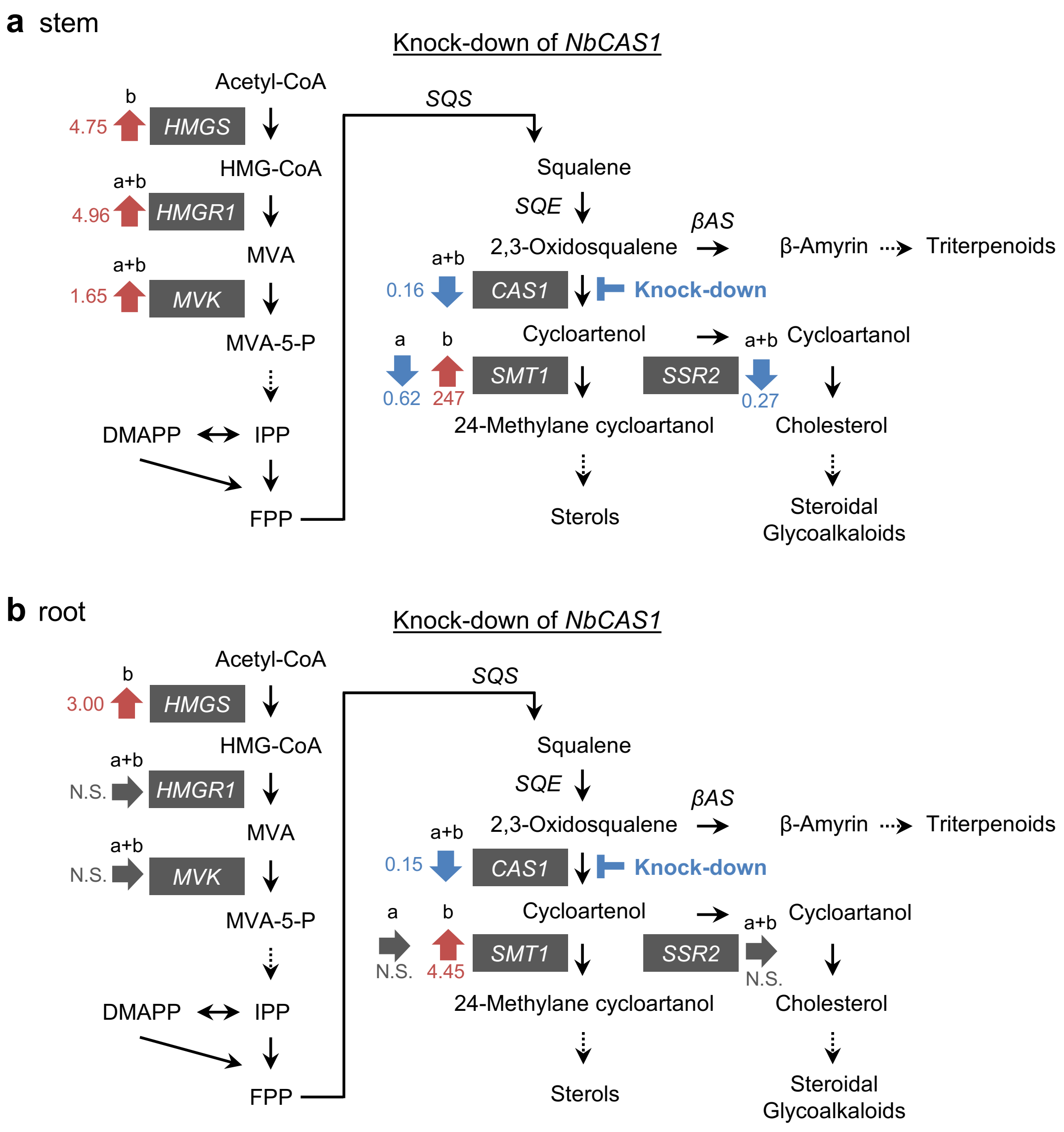

Supplementary Figure S4. Feedback and feed-forward regulation in isoprenoid biosynthetic genes in *NbCAS1* knockdown stems and roots. Schematic representation of fold changes in transcript levels of genes relative to the vector control (asGFP) in the *NbCAS1* knockdown stems (a) and roots (b) in isoprenoid biosynthetic pathway. Abbreviations of genes are described in the legend of Fig. 2. N.S. = not significant.

Supplementary Table S1 Isoprenoid biosynthetic genes used in this study

Gene		Abbreviation in this study	database*		cloned
			Gene ID	ORF length (nt)	ORF length (nt)
HMGS	3-hydroxy-3-methylglutaryl-CoA synthase	NbHMGSa	Niben101Scf01111g01003.1	1389	1389
		NbHMGSb	Niben101Scf01729g01015.1	1224	1389
HMGR1	3-hydroxy-3-methylglutaryl-CoA reductase 1	NbHMGR1a	Niben101Scf09686g00013.1	1818	1818
		NbHMGR1b	Niben101Scf13180g01003.1	1257	1818
HMGR2	3-hydroxy-3-methylglutaryl-CoA reductase 2	NbHMGR2a	Niben101Scf02203g05002.1	1989	1989
MVK	mevalonate kinase	NbMVKa	Niben101Scf25893g00005.1	948	1158
		NbMVKb	Niben101Scf00370g03023.1	633	1158
CAS1	cycloartenol synthase 1	NbCAS1a	Niben101Scf16532g01001.1	2220	2274
		NbCAS1b	Niben101Scf08080g00009.1	2220	2274
SSR2	sterol side chain reductase 2	NbSSR2a	Niben101Scf03969g04003.1	1710	1710
		NbSSR2b	Niben101Scf00271g04029.1	1710	1710
SMT1	S-adenosyl-L-Met-dependent C-24 sterol methyltransferase 1	NbSMT1a	Niben101Scf13874g01006.1	1041	1041
		NbSMT1b	Niben101Scf03085g05002.1	1014	1050

* Sol Genomics Network (https://solgenomics.net/)

Supplementary Table S2 Sequence identities between a and b of each gene

	nucleotide		amino acid		
	identity (%)	gap (%)	identity (%)	similarity (%)	gap (%)
NbHMGS	97.0	0.0	97.0	98.1	0.0
NbHMGR1	97.2	0.0	97.2	98.8	0.0
NbMVK	96.1	0.0	96.6	97.9	0.0
NbCAS1	98.1	0.0	97.9	99.3	0.0
NbSMT1	87.5	1.2	89.4	95.4	0.9
NbSSR2	96.5	0.0	96.5	98.6	0.0

Supplementary Table S3 Primers used for isolation of isoprenoid biosynthetic genes

gene	primer		
		#	sequence (5'-3')
NbHMGSa	Fw	670	CGACTTAGAGAAAGAAACAGTG
	Rv	671	ATTGAATTTGTTGAAAAATACT
NbHMGSb	Fw	672	AGAGAGAAAAAAAGCCGTTAC
	Rv	673	GTTGAAAACTAAAAAGGCATTG
NbHMGR1a	Fw	37	TCACTGCTTAATACTTCTTTCC
	Rv	38	CATTTCTTTAATAATGATGGTTC
NbHMGR1b	Fw	37	TCACTGCTTAATACTTCTTTCC
	Rv	38	CATTTCTTTAATAATGATGGTTC
NbHMGR2a	Fw	39	AGCGGGAAGCAGCCGGTACG
	Rv	40	GTAGCTACTTGTTGGAGCAC
NbMVKa	Fw	678	TCTCCGCCATAACCAACAAGGC
	Rv	679	ATATCCATATTCAAGAATGCAG
NbMVKb	Fw	680	CGCCATAACTAACCATCACGGT
	Rv	681	CCATATCGAAAGATCTTATTAG
NbCAS1a	Fw	65	TTCGTCTACCCATTTCTGAAG
	Rv	66	CCACGAAAGACGATTAGTTGC
NbCAS1b	Fw	81	CAGTCAGAGTGAATCTGTAGAAG
	Rv	68	AATAAAGTAAACTAGCTAAT
NbSSR2a	Fw	75	TGTACTGGCCTCTTTTTCCAAG
	Rv	42	GATCCATGTGAACCATTATTTG
NbSSR2b	Fw	43	AGATTTCTAGTTCTAACTAC
	Rv	44	AACACGAAACGGAACCGGAA
NbSMT1a	Fw	45	CTGCTTTCTACGATCCGTCTCTG
	Rv	46	ATGTGTAAAGTTCTGTGCGAAG
NbSMT1b	Fw	47	CATATTCTAATTATTCTGTATC
	Rv	48	AAATGTGTCCGACATGCAACAC

Supplementary Table S4 Primers used for construction of TRV vector

construct	primer		
		#	sequence (5'-3')
pTRV2/asNbPDS400	Fw	142	actgaattcATTGTACTGCCATTATTCAG
	Rv	141	actggatccTCAAATTTGCTATTGGACTC
pTRV2/asNbPDS200	Fw	257	actgaattcAGCAATCAAAATGCACTGCATC
	Rv	141	actggatccTCAAATTTGCTATTGGACTC
pTRV2/asNbHMGR1	Fw	132	actgaattcCCTCAATAATAAACTGCTCAG
	Rv	131	actggatccATGGACGTTCGCCGGCGACCT
pTRV2/asNbCAS1	Fw	134	actgaattcGAGCTTTTTCTCTCAATCTT
	Rv	133	actggatccGCCATTACTTCATGGGGGAA
pTRV2/asNbSSR2	Fw	136	actgaattcGCTTCCTTCAATCCCGAAGC
	Rv	135	actggatccTTACTTCTCCATATATCTAG
pTRV2/asNbSMT1	Fw	138	actgaattcATTCATTATTGTTGAGGCCT
	Rv	151	actggatccATGTCAAAACAAGGGGCTTTTG
pTRV2/asNbGFP400	Fw	144	actgaattcCGTCCTCCTTGAAATCGATTC
	Rv	143	actggatccATGAGTAAAGGAGAAGAACT
pTRV2/asNbGFP200	Fw	258	actgaattcCCATAAGTGAAAGTAGTGAC
	Rv	143	actggatccATGAGTAAAGGAGAAGAACT

Supplementary Table S5 Primers and probes used for real-time PCR

gene	primer			probe#*
		#	sequence (5'-3')	
NbHMGSb	Fw	746	AGCCGAAGAATGTCGGAAT	152
	Rv	747	CTGTGGGAGGCAAGTAGGAG	
NbHMGR1ab	Fw	318	AACTATGCCTTCTATTGAGGTTGG	119
	Rv	319	GCTGATTGTGATGCAAGTTGA	
NbMVKab	Fw	770	ATGGGTGTTAGCCATGCTTC	75
	Rv	771	TTCCTGACAGAAGGGTTGGT	
NbCAS1ab	Fw	170	CATCATGGGCTATGCTGACTC	120
	Rv	171	GTGTAATGGCGTGGGATTTC	
NbSMT1a	Fw	751	GCTGCAGAAGGTCTTGTCG	142
	Rv	752	TTGCGAACCACGAAGAAGTA	
NbSMT1b	Fw	778	GCTGGTTTCGAAGTTGTATGG	73
	Rv	779	AGGCAAGTACCACGGAAGAG	
NbSSR2ab	Fw	172	CACCGCGAAATGGAGGTAT	21
	Rv	173	CAGGCAGCTTGTAAATTCTGTG	
NbEF1α	Fw	179	CTGGTACCTCCCAAGCTGAC	56
	Rv	180	CCAGCTTCAAAACCACCAGT	

*UPL universal probe library (Roche Diagnostics)
